# Relevance of Viroporin Ion Channel Activity on Viral Replication and Pathogenesis

**DOI:** 10.3390/v7072786

**Published:** 2015-07-03

**Authors:** Jose L. Nieto-Torres, Carmina Verdiá-Báguena, Carlos Castaño-Rodriguez, Vicente M. Aguilella, Luis Enjuanes

**Affiliations:** 1 Department of Molecular and Cell Biology, National Center of Biotechnology (CNB-CSIC), Campus Universidad Autónoma de Madrid, 28049 Madrid, Spain; E-Mails: jlnieto@cnb.csic.es (J.L.N.-T.); ccastano@cnb.csic.es (C.C.-R.); 2 Laboratory of Molecular Biophysics, Department of Physics, Universitat Jaume I, 12071 Castellón, Spain; E-Mail: verdia@uji.es

**Keywords:** viroporins, virus, ion channel, protein-lipid pore, replication, pathogenesis, inflammasome

## Abstract

Modification of host-cell ionic content is a significant issue for viruses, as several viral proteins displaying ion channel activity, named viroporins, have been identified. Viroporins interact with different cellular membranes and self-assemble forming ion conductive pores. In general, these channels display mild ion selectivity, and, eventually, membrane lipids play key structural and functional roles in the pore. Viroporins stimulate virus production through different mechanisms, and ion channel conductivity has been proved particularly relevant in several cases. Key stages of the viral cycle such as virus uncoating, transport and maturation are ion-influenced processes in many viral species. Besides boosting virus propagation, viroporins have also been associated with pathogenesis. Linking pathogenesis either to the ion conductivity or to other functions of viroporins has been elusive for a long time. This article summarizes novel pathways leading to disease stimulated by viroporin ion conduction, such as inflammasome driven immunopathology.

## 1. Introduction

Cells maintain optimum subcellular compartment ionic conditions, different to those of the extracellular media by controlling ion transport through lipid membranes. Different cell organelles present particular ion compositions. These asymmetric distributions of ions among biological membranes generate electrochemical gradients, essential for the proper cell functioning [1]. Crucial aspects of the cell are governed by the membrane potential, Ca^2+^ stores in the endoplasmic reticulum (ER) and the Golgi apparatus, and different pH conditions found in the organelles of the secretory pathway, which benefit from those ion gradients. The coordinate action of a multitude of ion channels and transporters generates and tightly controls these ionic milieus found within cells.

It is well known that viruses exploit and modify host-cell ion homeostasis in favor of viral infection. To that purpose, a wide range of viruses encode viroporins [2]. Viroporins constitute a large family of multifunctional proteins broadly distributed in different viral families, and are mainly concentrated in RNA viruses [2]. Highly pathogenic human viruses, such as influenza A virus (IAV), human immunodeficiency virus 1 (HIV-1), hepatitis C virus (HCV), several picornaviruses, respiratory syncytial virus (RSV), and coronaviruses (CoVs), such as the one responsible for the severe acute respiratory syndrome (SARS-CoV), and the etiologic agent of Middle East respiratory syndrome (MERS-CoV), encode at least one viroporin [3,4,5,6,7,8,9]. These are transmembrane proteins that stimulate crucial aspects of the viral life cycle through a variety of mechanisms. Noticeably, these proteins oligomerize in cell membranes to form ion conductive pores, which generally display mild ion selectivity, indicating that viroporins do not show preference for particular ionic species. The measurements of channel conductance are in accordance with the formation of relatively wide pores, supporting the non-specificity of viroporins. The influence of the lipid charge in channel function is a distinctive feature of some viroporins, as reported in the case of SARS-CoV E protein [10,11]. Ion channel (IC) activity is relevant for virus propagation and may have a great impact on host-cell ionic milieus and physiology [2,12,13]. Once inserted on cell membranes, viroporins tune ion permeability at different organelles to stimulate a variety of viral cycle stages that will be described below. IC activity ranges from almost essential, to highly or moderately necessary for viruses to yield properly.

Besides modifying cellular processes to favor virus propagation, the loss of ion homeostasis triggered by viral IC activity may have deleterious consequences for the cell, from stress responses to apoptosis [2,14,15]. That is why cells have evolved mechanisms to sense the ion imbalances caused by infections and elaborate immune responses to counteract viruses. Interestingly, the IC activity of several viroporins triggers the activation of a macromolecular complex called the inflammasome, key in the stimulation of innate immunity [16,17,18,19,20,21]. Inflammasomes control pathways essential in the resolution of viral infections. However, its disproportionate stimulation can lead to disease. In fact, disease worsening in several respiratory viruses infections is associated with inflammasome-driven immunopathology [22,23].

Taking into consideration the relevance of IC activity in viral production, and its direct effect in pathology and disease, ion conductivity and its pathological stimulated pathways can represent targets for combined therapeutic interventions.

## 2. Ion Channels Formed by Viroporins

In general, viroporins are small proteins (less than 100 amino acids) with at least one amphipathic helix that constitutes its transmembrane domain, spanning lipid membranes [2]. Larger viroporins have also been described in CoVs. This is the case of SARS-CoV 3a protein, porcine epidemic diarrhea virus (PEDV) 3 protein, or human coronavirus 229E (HCoV-229E) 4a protein [24,25,26]. To form the ion conductive pore, viroporins self-assemble and oligomerize, which is a key feature of this family of proteins. Structural studies for either the transmembrane domain or for full-length viroporins have revealed the molecular architecture of these viral ion channels, which can present different oligomerization statuses. IAV M2, picornavirus 2B and Chlorella virus Kcv form tetrameric structures [27,28,29,30], whereas pentamers have been described for HIV-1 Vpu, SARS-CoV and MERS-CoV E proteins, and RSV SH protein [9,31,32,33,34]. HCV p7 and human papillomavirus E5 proteins form hexameric channels [35,36]. In addition, most measurements of channel conductance are in accordance with the formation of relatively wide pores, again in agreement with the non-specificity of viroporins [6,10,37,38].

Ion selectivity of ion channels indicates the preference of the pore for a specific ion and defines the functional roles that the channel may display. It is known that IAV M2 protein channels are highly selective for protons and ion conductance is activated at low pH [39,40,41]. Likewise the IAV M2 channel, the Kcv protein of Chlorella virus is another highly selective channel, which contains a conserved K^+^ selectivity filter [29]. Nevertheless, most viroporins usually show mild ion selectivity, which means that, in general terms, these channels do not display preference for a particular ion. HIV-1 Vpu protein displays a mild cationic selectivity in NaCl and KCl electrolyte solutions [4]. Similarly, HCV p7 channels are selective for monovalent cations (Na^+^ and K^+^) over monovalent anions (Cl^−^) [42]. Moreover, functionally relevant H^+^ transport has also been identified for this protein in cell culture [12]. ORF4a protein of HCoV-229E forms a channel that prefers cations over anions but does not show a clear specificity for a particular type of cation [25]. Still, a lot of electrophysiology experiments remain to be done for a proper characterization of viroporin selectivity. Setting aside a few highly proton selective viroporins, the vast majority display a weak selectivity, either cationic or anionic, which is strongly dependent on the lipid charge of their host membrane, as discussed below in detail.

**Figure 1:**
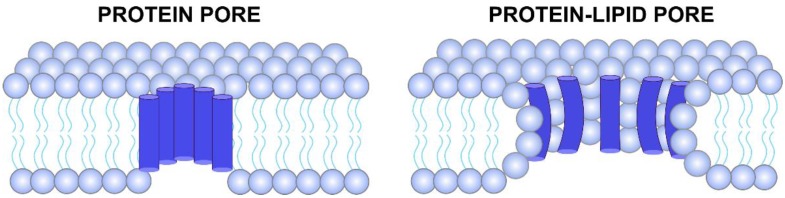
Ion channels formed by viroporins. Left depiction represents a channel exclusively formed by protein monomers (blue cylinders) inserted on a lipid membrane. Schematic on the right shows a protein-lipid pore. In this latter case, the lipid head groups (cyan circles) are oriented towards the channel pore, modulating ion conductance and selectivity.

Interestingly, under some circumstances the selectivity of these channels can be modulated. SH protein exhibits a poor cationic selectivity at neutral pH, which turns into anionic at acidic pH [43]. This is consistent with the titration of histidines, the only titratable residues of the SH protein. The presence of Ca^2+^ in selectivity experiments performed in KCl solutions reduced the cationic preference of p7 channels, which may indicate that Ca^2+^ affect the selectivity filter [42]. Probably, the most striking mechanism influencing IC selectivity and conductance has been recently reported for SARS-CoV E protein [10,11]. It was observed that the lipids are an integral component of the pore structure because electrophysiological measurements proved that the lipid charge modulates the ion transport properties of the SARS-CoV E protein. These findings suggested that viroporins can assemble into alternative complex structures, forming protein-lipid pores (Figure 1).

**Figure 2:**
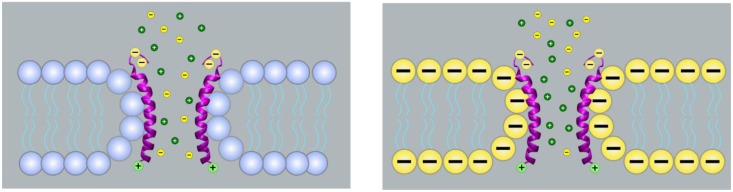
Functional involvement of lipid head groups in the protein-lipid pore formed by SARS-CoV E protein. Depictions represent E protein channels inserted in non-charged membranes (left) or negatively-charged membranes (right), under low solute concentrations and neutral pH. In these circumstances, each E protein monomer presents two negative charges provided by glutamic acid residues, and a positive charge conferred by an arginine. When reconstituted in fully or partially negatively-charged membranes, lipid head groups provide additional negative charges to the pore, which makes E protein channel more selective for cations and more conductive.

The IC activity of a number of transmembrane proteins, as well as of small peptides and antimicrobial peptides, is strongly dependent on the lipid environment. The case of SARS-CoV E protein is a clear example of how lipid membrane charge influences the main ion transport properties of an IC. E protein behaves quite differently when reconstituted in neutral or negatively-charged membranes (Figure 2).

Conductance experiments showed that in non-charged phosphatidylcholine membranes, the E protein channel acts as a non-selective neutral pore, since the channel conductance changes linearly with bulk solution conductivity [11]. In contrast, in negatively charged phosphatidylserine membranes the variation of channel conductance with solution concentration follows different regimes depending on the salt concentration; a characteristic trait of charged pores [10,11,44]. These experiments, together with ion selectivity measurements [11], suggest that E protein behaves as a charged pore in negatively-charged membranes. Parallel observations are made when reconstituting SARS-CoV E protein in membranes containing a small percentage of charged lipids and, in particular, in membranes containing the same amount of charged lipid (~20%) as the ERGIC-Golgi membranes, where E protein channel is presumably localized [10]. These observations support the view that some polar lipid heads line the pore lumen together with the protein transmembrane helices and that both, protein and lipids, contribute to channel selectivity. An additional proof reinforcing this hypothesis is provided by electrophysiology experiments using lipids with negative intrinsic curvature. It is known that the protein-lipid pore formation is favored in membranes with positive curvature [45]. In fact, when E protein is reconstituted in dioleoyl phosphatidylethanolamine membranes, a lipid with intrinsic negative curvature, it is much more difficult to achieve channel insertion because the membrane negative curvature becomes energetically unfavorable for the assembly of a proteolipidic structure [10]. The E protein-lipid channel may not constitute a unique case, as poliovirus 2B permeabilization displayed a strict requirement for anionic phospholipids in the membrane composition [28], which may indicate that lipid molecules are involved in the pore formation.

Regulatory mechanisms, such as gating, operate in some viroporins. Histidine residues present in the IAV M2 transmembrane domain become protonated when encountering low pH conditions, and experience a conformational change that opens the channel pore allowing H^+^ flux [46]. The influence of the histidine residues in the IC is also observed in the SH protein. In this protein the conductance is pH-dependent and consistent with the titration of these residues [43].

Viroporins could be regulating the transport capacity of cellular ion transporters, besides showing their intrinsic IC activity. A well-defined case is that of human papillomavirus oncoprotein E5, which interacts with vacuolar H^+^ ATPase (V-ATPase) modulating its function and leading to cell transformation [47,48]. Interestingly, other viroporins interact with cellular ion pumps. SARS-CoV E protein binds the alpha subunit of Na^+^/K^+^ ATPase in infected cells [49]. IAV M2 protein binds Na^+^/K^+^ ATPase beta1 subunit [50]. It is well known that the ion transport capacity of cellular pumps such as Na^+^/K^+^ ATPase and sarcoendoplasmic reticulum Ca^2+^ ATPase (SERCA) may be influenced by the interaction with other proteins. FXYD proteins and phospholamban are well-characterized examples [51,52]. These regulatory proteins are small transmembrane proteins that oligomerize and eventually form ICs when reconstituted in artificial lipid membranes [53], as happens for viroporins. Some examples of viroporins modifying the activity of cellular ion channels have also been reported. HIV-1 Vpu interacts with the K^+^ channel TASK-1 promoting viral release [54]. In addition, indirect inhibition of epithelial sodium channels has been reported for IAV M2 and SARS-CoV E proteins [55,56]. The possibility of a dual role of ion modulation by viroporins, depending both on their intrinsic transport properties and on their possible regulatory activity on key cellular ion transporters, increases the potential relevance of this family of proteins.

## 3. Ion Channel Activity and Virus-Host Interaction

### 3.1. Virus Production

Viroporins are involved in processes relevant for virus production. In general, these proteins do not affect viral genome replication, but stimulate other key aspects of the viral cycle such as entry, assembly, trafficking and release of viral particles [2,13]. As a consequence, partial or total deletion of viroporins usually leads to significant decreases in viral yields. HCV p7 viroporin is indispensable for virus propagation, as no infectious viruses are recovered when p7 protein is eliminated [57]. Other viruses are more tolerant to viroporin deletion. Thus, IAV or HIV-1 lacking M2 and Vpu, respectively, can be efficiently rescued. Nevertheless, in general, viroporin defective viruses show significantly lower virus yields, ranging from 10- to 100-fold [58,59]. The relevance of E viroporin in coronavirus production seems to be species-specific. Deletion of E gene in transmissible gastroenteritis virus (TGEV) and MERS-CoV generates replication-competent propagation-deficient viruses [60,61]. E gene is not essential for virus production in SARS-CoV and mouse hepatitis virus (MHV), although in its absence viral titters are reduced from 100- to 1000-fold, respectively [62,63]. Silencing of SARS-CoV 3a protein, PEDV 3 protein, and HCoV-229E 4a protein expression in virus infection cause a reduction in virus titers [24,25,64], supporting that these viroporins are involved in virus production. In the case of SARS-CoV 3a protein, this has also been shown in mice infected with a SARS-CoV variant missing 3a protein (C. Castaño-Rodriguez, J.L. Nieto-Torres and L. Enjuanes, unpublished results). In some cases, viroporin deletion induces a growth restriction that can be cell or tissue specific. Thus, RSV lacking the SH gene, shows an efficient growth in cell culture, but a limited production in the nasal turbinates of infected mice or chimpanzees, as compared with the wild type virus [65,66].

Collectively, previous data support that viroporins stimulate virus propagation. A key issue is to understand the relevance of their IC conductance on this activity. Both viroporin IC activity and other functions not related to ion conductivity apparently affect virus propagation. Some non-conductive viroporin domains are important players in viral morphogenesis. The cytoplasmic tail of several viroporins actively participates in virus production. A few examples are now briefly described. CoV E protein C-terminal domain interacts with the viral membrane (M) protein during the morphogenesis process [67]. In fact, the last nine amino acids of SARS-CoV E protein C-terminus stimulate virus growth [68]. This protein sequence includes a PDZ-binding motif involved in interactions with cellular proteins and virulence. IAV M2 cytoplasmic domain interacts with the M1 protein to favor virus assembly at the site of budding [69]. An amphipathic helix located in the cytoplasmic tail of the IAV M2 protein induces bending of cellular membranes, necessary for budding, and excision of the nascent particles [70]. This functional domain works independently to the M2 ion conductive properties. HIV-1 Vpu also facilitates budding termination of newly formed viruses in an IC independent manner. Vpu deleted viruses are less efficiently released and remain attached to the plasma membrane of infected cells [59]. In this case, the transmembrane domain of Vpu accounts for this phenotype by establishing critical interactions with cellular proteins such as one with the interferon-stimulated protein tetherin. This binding prevents tetherin from inhibiting the release of viral particles at the plasma membrane of infected cells [71,72]. Both IC activity and tetherin inhibitory property are concentrated in the Vpu transmembrane domain, nevertheless these functions work independently [73].

Accumulating reports support that viroporin ion conductivity favors virus yields. Inhibition of viroporin IC properties, through subtle mutations that may not interfere with other functions of the protein, affects viral growth to different extents. Point mutations that blocked HCV p7 IC activity either completely abrogated virus production or resulted in a 100-fold restriction in virus yields, depending on the virus strain [12,57]. Influenza viruses lacking M2 ion conductivity, presented either a 15-fold reduction of viral titer in tissue culture [74], or showed a standard production in cell culture but a restricted growth in the nasal turbinates of infected mice [75]. SARS-CoV E protein ion conductivity also supports viral production and fitness. E protein IC activity was knocked down by introducing point mutations in key residues of its transmembrane domain [10]. Although not essential for virus production, E protein IC activity stimulated viral propagation. Viruses lacking E protein ion conductivity were outcompeted by others displaying this function [22]. In fact, IC defective viruses tended to restore ion conductance by introducing compensatory mutations in the transmembrane domain of the protein both in cell cultures and in mice [22]. In agreement with these data, IAV lacking M2 IC activity showed fitness defects, as it was outgrowth by the parental virus, in an even faster manner than that observed in SARS-CoV [74]. The relevance of ion conductivity in boosting virus production remains unknown for the moment in other viral systems. Initial findings argued that scrambling of the HIV-1 Vpu transmembrane domain inactivated ion conductivity and partially inhibited viral production [76]. However, this alteration of the transmembrane domain affected the Vpu-tetherin interaction and IC activity. Recent reports showed that mutations that specifically inhibited ion conductivity, but not Vpu-tetherin interaction, did not affect VLP production; therefore, this interaction with tetherin seems to be responsible for the observed phenotype [73].

Pharmacological inhibition of viroporin ion conductance further supports the role of IC in virus propagation. Several compounds inhibit viroporin ion conductivity in artificial lipid membranes, and some of them efficiently reduce viral growth when administered to infected cells. Inhibition of M2 protein IC activity mediated by amantadine prevents or decreases viral growth in a strain specific manner [39,77]. Amantadine also inhibits HCV growth [13]. HCoV-229-E and MHV growth is restricted by hexamethylene amiloride, which blocks E protein ion conductivity [78]. The viral growth inhibitory properties of these compounds encourage its use as potential antivirals. This aspect will be further addressed below.

**Figure 3:**
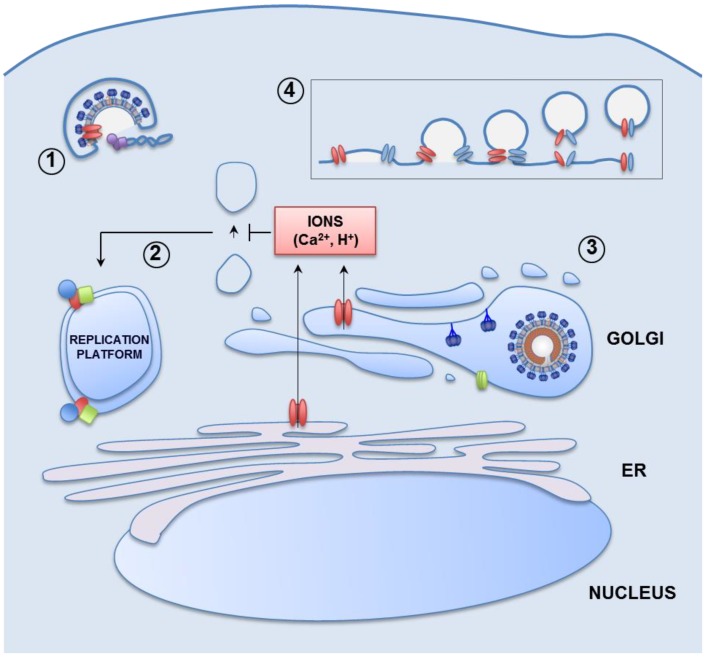
Pathways stimulated by viroporin ion channel activity leading to virus production. Viral-membrane embedded viroporins (red ellipses) transport H^+^ inside the endocytosed virion. This causes structural changes in fusion and matrix proteins facilitating the uncoating of viral ribonucleoproteins (**1**); Viroporin-mediated ions leak from intracellular organelles such as the endoplasmic reticulum (ER) or the Golgi apparatus towards the cytoplasm causes a blockade of vesicle transport and/or hijacking of autophagic membranes. These processes finally result in the accumulation of membranous structures that will serve as platforms for viral replication and morphogenesis. Blue, red and green structures show the viral replicase (**2**); In addition, equilibration of Golgi and secretory pathway organelles’ pH protect both viral proteins involved in entry (blue structures and green ellipses) and newly formed virions that can be sensitive to acidic environments (**3**); Viroporins (red and blue ellipses), locate in the budding neck of some enveloped viruses. These proteins may interact and oligomerize, rearranging the formation of channels, which additionally could facilitate virion scission (**4**).

Widely conserved pathways influenced by viroporin ion conductivity, and others that seem to be species-specific are now described and graphically summarized to facilitate their overview (Figure 3).

#### 3.1.1. Viral Entry

Early key aspects of virus cycle, such as viral entry, can be boosted through ion conductivity. Non-enveloped viral particles lack viroporins as structural components, and these proteins are poorly represented in the membrane of enveloped viruses [63,79,80,81]. Nevertheless, the viroporin molecules embedded in the viral envelope can actively participate in viral entry; IAV M2 protein is a well characterized example. IAV is internalized within the cell through an endocytic process. Endosomes containing viral particles fuse with acidic organelles to form endolysosomes. The H^+^ ATPase of these organelles pumps H^+^ inside their lumen lowering the pH. The few M2 copies present in the IAV envelope are activated by the low pH conditions and allow the flux of H^+^ inside the viral particle. The acidification of the endocytosed virion drives a series of conformational changes in the viral hemagglutinin (HA) protein, leading to the exposure of a fusion peptide [82,83]. In parallel, the M1 protein layer, which underlies the viral envelope, and protects the viral ribonucleoproteins, is disassembled under these pH conditions [84]. The fusion peptide finally triggers the fusion of the viral and endosomal membranes, resulting in the release of viral ribonucleoproteins in the cell cytoplasm. Inhibition of M2 protein IC activity by amantadine results in incomplete uncoating of the virion in some IAV strains [39,77]. This mechanism does not seem relevant for HCV virus, as its entry is independent of p7 IC activity [12]. Whether or not this pathway, or related ones, participate in the entrance of other viral species remains to be established.

#### 3.1.2. Remodeling of Cell Organelles

Modification of the ionic content of intracellular compartments can ultimately result in functional and morphological transformations. The ion conductivity of several viroporins affects protein transport. Alteration of the ionic content of the Golgi apparatus and the endosomes interfere with cellular protein processing and sorting [85]. IAV M2 causes a lag in protein transport through the Golgi apparatus [86]. This delay on transport can be inhibited by the addition of amantadine, an IC activity inhibitor of M2 protein. Furthermore, monensin, an H^+^/Na^+^ antiporter, causes similar defects. Both M2 and monensin induce dilation of Golgi cisternae, although to different extents. Infectious bronchitis virus (IBV) E protein also affects protein transport through the secretory pathway, whereas the mutant predicted to inhibit IC activity does not [87]. Coxsackievirus 2B protein disturbs pH and calcium homeostasis in the Golgi and the ER, thereby inhibiting protein transport. Mutations inhibiting 2B ion conductivity restored proper protein trafficking [88]. Ca^2+^ is involved in membrane fusion events, and its leakage to the cell cytoplasm causes anterograde vesicle trafficking blockage. SARS-CoV 3a protein is both necessary and sufficient for SARS-CoV Golgi fragmentation and promotes the accumulation of intracellular vesicles that may be used for virus formation or for non-lytic release of virus particles [89]. The exact relevance of protein transport delay on virus production remains to be established. It has been speculated that alteration of ionic homeostasis in transport vesicles affects anterograde trafficking leading to conglomeration of cell membranes that will serve as the platforms for viral replication and budding [88]. In addition, ionic efflux from intracellular organelles can lead to pathways triggering the accumulation of autophagic vacuoles as platforms for virus replication. In fact, rotavirus nsp4 viroporin allows Ca^2+^ efflux form ER thereby triggering autophagy in favor of viral infection [90].

#### 3.1.3. Protection of the Viral Progeny

In many cases, viral proteins, and even virions, have to progress through the secretory pathway, encountering the low pH found within the Golgi apparatus lumen [91]. These acidic conditions may inactivate forming virions, which eventually are acid-sensitive [12]. Viroporin ion conductivity is critical in viral progeny protection and, occasionally, it can be trans-complemented by heterologous viroporins. IAV M2 IC activity is thought to keep the pH of the Golgi apparatus and its associated vesicles above a threshold, in order to avoid conformational changes in HA which may lead to its premature activation rendering non-infectious viruses. In fact, blocking of M2 ion conductivity by amantadine induces irreversible activation of HA [92]. This effect can be overcome by the treatment of infected cells with monensin that alkalizes the Golgi in an analogous manner to M2 protein. Similarly to what has been shown for M2 protein, HCV p7 protein mediates a H^+^ leak from intracellular organelles, and this alkalinization is required for the production of infectious viruses [12]. Mutant viruses lacking p7 ion conductivity were not rescued, and treatment with amantadine greatly diminished the production of wild type infectious virions. Furthermore, in the absence of p7 IC activity, alternative approaches to balance the pH of intracellular organelles result in partial recovery of infectious viruses. Either Bafilomycin A1 treatment, an inhibitor of a H^+^ vacuolar ATPase (V-ATPase), or expression of IAV M2, but not the M2 IC mutant, rescued virus production [12].

#### 3.1.4. Release of Newly Formed Virions

The increase of cellular permeability by viroporins, IC activity may ultimately result in cell lysis, a requisite for the egress of non-enveloped viruses [2]. It has also been speculated that disruption of ion gradients may trigger the membrane fusion events necessary for budding termination and release of enveloped viruses [2]. Nevertheless, in the latter case other non-ion conductive viroporin functions, as those previously described for M2 and Vpu, may also be relevant in the release process. In addition, we propose that viroporins, located in the budding neck of forming viruses [70], could oligomerize to form an IC facilitating membrane fusion and budding termination.

### 3.2. Pathogenesis

Besides their key role in virus propagation, viroporins are also virulence factors in different viral systems. Total or partial deletion of non-essential viroporins usually leads to attenuated phenotypes. Interestingly, these viroporin-deleted viruses have been successfully used as potential vaccine candidates. Mice infected with an IAV lacking M2 protein showed no weight-loss, nor pathology, and were protected against the wild type virus [93]. An RSV defective for SH protein showed growth attenuation in the upper respiratory tracts of mice and chimpanzees and caused mild disease [65,66]. A classical swine fever virus (CSFV) missing full-length or partial-length p7 protein, similar to HCV p7 viroporin, lacked virulence [94]. A SARS-CoV lacking the full-length E protein (SARS-CoV-ΔE) was attenuated in hamsters, transgenic mice expressing the human SARS-CoV receptor (hACE2), and in both young and elderly mice. SARS-CoV-ΔE induced increased stress and limited NF-κB driven inflammation [62,95,96]. In addition, elimination of defined E protein regions of 6–12 amino acids in its carboxy-terminus leads to viral attenuation [68,97]. Several of these mutants are promising potential vaccine candidates for SARS-CoV. SARS-CoV 3a protein regulates host-cellular responses involved in the activation of pro-inflammatory genes such as C-Jun and NF-κB [98,99,100], and in the production of pro-inflammatory cytokines and chemokines such as IL-8 or CCL5 [99]. Furthermore, 3a protein IC activity has been linked to its pro-apoptotic function [101]. PEDV 3 protein has also been involved in pathogenesis as a PEDV with a 49-nucleotide deletion in 3 gene lacks IC activity and is attenuated [24].

**Figure 4:**
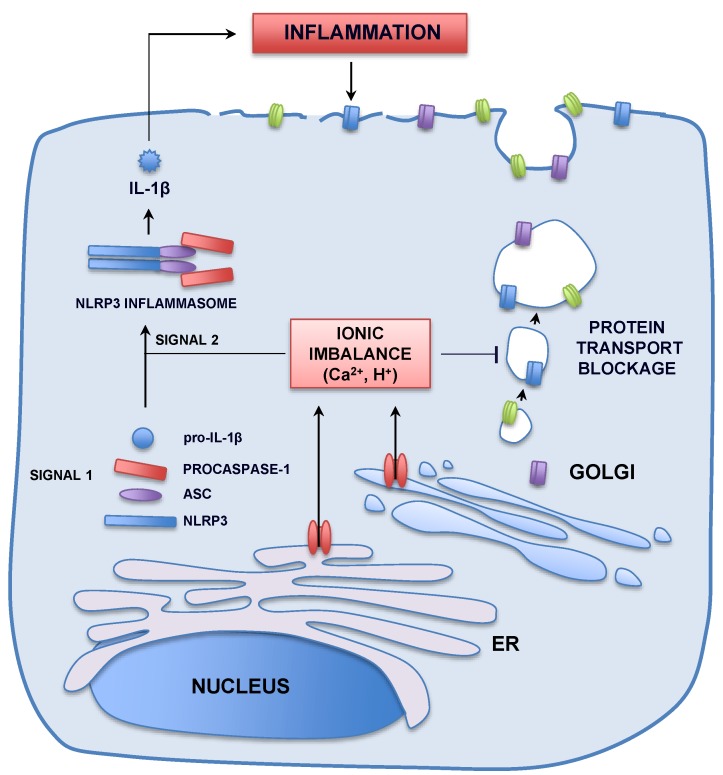
Pathways stimulated by viroporin ion channel activity leading to pathology. Molecular patterns associated with viral infections are recognized by cellular sensors (signal 1), which activate the transcription and translation of the NLRP3 inflammasome components (NLRP3, ASC and procaspase-1) and the inactive pro-IL-1β. Viroporins inserted in the intracellular organelles, such as the endoplasmic reticulum (ER) or the Golgi apparatus, favor the leak of Ca^2+^ and H^+^ ions that move following their electrochemical gradient into cell cytoplasm. This ionic imbalance (signal 2) induces the assembly of the inflammasome complex, which triggers the maturation of pro-IL-1β into IL-1β through the action of caspase-1. Secreted IL-1β mediates a potent pro-inflammatory response that can be deleterious for the cell and the organism, when overstimulated. In addition, alteration of ionic milieus in intracellular compartments comes along with a protein transport delay or blockage. This results in a decrease of the levels of MHC-I molecules (blue rectangles) at the plasma membrane, preventing the infected cell to be recognized by the immune system. Protein transport blockage also diminishes the levels and activity in the cell surface of ion channels and transporters, crucial in the resolution of edema accumulation. Epithelial sodium channels (green structure) and Na^+^/K^+^ ATPase (purple rectangles) impairment have been related to the worsening of viral respiratory diseases such as those caused by SARS-CoV, IAV or RSV.

Viroporins are frequently associated with virus propagation, as their deletion from the viral genome usually causes viral titer drops that, by themselves, could contribute to the attenuated viral phenotype. However, viroporin removal can modulate cellular signaling pathways leading to virulence. It has been proposed that the protein transport blockage exerted by the IC activity of viroporins, such as that from coxsackievirus 2B and coronavirus E proteins causes a delay in the transport of major histocompatibility complex class I (MHC-I) molecules towards the plasma membrane [87,88,102]. This MHC-I trafficking defect allows the evasion of adaptive immune responses, leading to more productive infections (Figure 4).

Over stimulation of immune responses can also lead to pathogenesis. Indeed, IC activity is an important trigger of immunopathology, as demonstrated for SARS-CoV E protein. Mutant viruses lacking ion conductivity, which showed proficient replication in mice lungs, presented an attenuated phenotype. Animals infected with the SARS-CoVs lacking E protein IC activity showed a reduced mortality in comparison with those inoculated with the parental virus [22]. Interestingly, mice infected with the IC defective viruses presented much less edema accumulation in the lung airways, the ultimate cause of acute respiratory distress syndrome (ARDS) [103]. Flooded bronchioles and alveoli fail to interchange gases, leading to hypoxemia and eventually to death. Noticeably, there was a good correlation between edema accumulation and the disassembly of the airway epithelia which, when intact, drive edema resolution through an ion mediated water reabsorption. Edema accumulation and airway epithelia damage is accompanied by an exacerbated proinflammatory response in the lung parenchyma [103]. Animals infected with the IC deficient viruses presented decreased levels of IL-1β, TNF and IL-6 in the lung airways, key mediators of the ARDS progression. IL-1β is a key orchestrator of the inflammatory response and plays a critical role in counteracting invading viruses, however overstimulation of this pathway can lead to unwanted deleterious effects for the organism. In fact, over production of IL-1β is related with important pathologies such as gout, atherosclerosis, diabetes, ARDS and asthma [104,105,106,107]. As a consequence, IL-1β production is tightly regulated in the organism, through the inflammasome multiprotein complex.

How can viral IC activity stimulate this exacerbated inflammatory response leading to pathology and disease? Inflammasomes participate in pathogen recognition by sensing disturbances in cellular milieus, including intracellular ionic concentrations [108,109]. The NLRP3 inflammasome is one of the most extensively studied [110]. Two signals are generally required to activate this complex: the first one is usually triggered by molecular patterns associated to the viral infection, such as double-stranded RNA. This leads to the transcription and translation of the different components of the inflammasome and the immature pro-IL-1β [16]. Only when a second signal is simultaneously present, the components of the inflammasome are assembled, leading to the cleavage and activation of caspase-1. This protein processes pro-IL-1β into its active form IL-1β that is released to the extracellular media to promote proinflammation [16]. Interestingly, viroporin IC activity represents the second signal required for inflammasome activation, inducing the release of active IL-1β (Figure 4). In general, viroporins induce an efflux of ions, such as H^+^ and Ca^2+^ that move from their intracellular stores into the cytoplasm following strong electrochemical gradients, triggering the NLRP3 inflammasome. IAV M2 was the first noticed ion conducting viral protein activating this pathway [16]. Nowadays, several other viroporins stimulating the inflammasome have been identified. RSV SH, human rhinovirus 2B, encephalomyocarditis virus 2B, HCV p7, and IAV PB1-F2, among others, are additional examples [17,18,19,20,23,111]. This mechanism of overstimulation of IL-1β production seems to have key consequences in pathogenesis. The severity of human rhinovirus infection is linked to overinflammation and release of cytokines, including IL-1β. Rhinovirus 2B protein causes a Ca^2+^ leakage from the ER and Golgi apparatus essential for inflammasome activation and IL-1β production [19]. Similarly, RSV SH activates this pathway resulting in immunopathology [18].

Given the wide variety of effects that viroporin IC activity has on viral propagation and its influence on pathogenesis, inhibition of IC conductivity has been a promising target for therapeutic interventions. A growing list of compounds interfering with viroporin IC activity of several viruses such as IAV, HCV, HIV-1, coronaviruses, and RSV, has been described [43,78,112,113,114]. Despite being active in artificial lipid bilayers, and sometimes in cell culture, the pharmacological use of antagonists of IC activity in humans is still limited to a reduced number of cases. Amantadine was the first inhibitor of viroporin IC activity approved for use in humans, and it has been utilized in clinics for around 20 years in the treatment of IAV [113]. Amantadine binds M2 protein at the N-terminal lumen of the channel and at the C-terminal surface of the protein with high and low affinities, respectively, blocking ion conductance and interfering with viral replication [115]. However, IAV variants containing mutations in the M2 transmembrane domain that conferred resistance to amantadine emerged [115,116]. Amantadine and hexamethylene amiloride are effective inhibitors of HCV p7 IC activity as shown using *in vitro* systems [42,112]. HCV shows a genotype-dependent sensitivity to amantadine, when the drug is applied at high doses, which may explain its inefficacy in infected patients, limiting the application of amantadine in HCV disease treatment [117]. Similarly, high concentrations of hexamethylene amiloride are required to inhibit p7 conductivity in cell culture, which increased its toxicity, making this drug unsuitable for clinical administration [117]. However, drug screenings have identified other promising compounds interfering p7 IC activity, such as long alkyl iminosugars derivatives and BIT225, with the latter showing modest but successful restriction of HCV load in infected patients [6,118]. These compounds may represent the basis for a therapeutic treatment of hepatitis C.

Taking into consideration the fast selection of drug-resistant viruses under drug pressure, the simultaneous inhibition of IC activity, and other signaling pathways stimulated by ion conductivity leading to pathology, may represent a better treatment option. Interfering with these pathological pathways constitutes a valuable approach, with the advantage that it is independent of the appearance of drug resistant viruses. Targeting exacerbated inflammatory responses, such as those triggered by IC activity leading to inflammasome activation in various respiratory infections, may reduce disease worsening and progression [23]. Novel specific inhibitors of NLRP3 inflammasome showing promising results for inflammatory diseases could be applied for these purposes [119].

## 4. Summary and Future Prospects

Viroporins have been known for a long time as key contributors to virus propagation and stimulators of pathogenesis. Several reports have dissected the crucial impact that IC activity has in these processes. It is remarkable how these small channels have high implications at the cell level favoring virus production and, at the same time, causing disturbances at a tissue or organism level eventually leading to pathology. Understanding the molecular and physicochemical structure of these ion pores may constitute the basis for rational design of specific IC activity inhibitors and strategies to counteract the pathology mediated by IC activity.
